# Supplementary material for: Neural circuit models for evidence accumulation through choice-selective sequences
Source: Nat Commun. 2026 Mar 17;17:4055. doi: 10.1038/s41467-026-70267-9 (PMC13139583; doi:10.1038/s41467-026-70267-9)
Supplement: Supplementary file 1 — Supplementary Information [file 41467_2026_70267_MOESM1_ESM.pdf]

SUPPLEMENTARY INFORMATION

Supplementary Figures 1-13
Supplementary Text providing more formal analysis of the models.

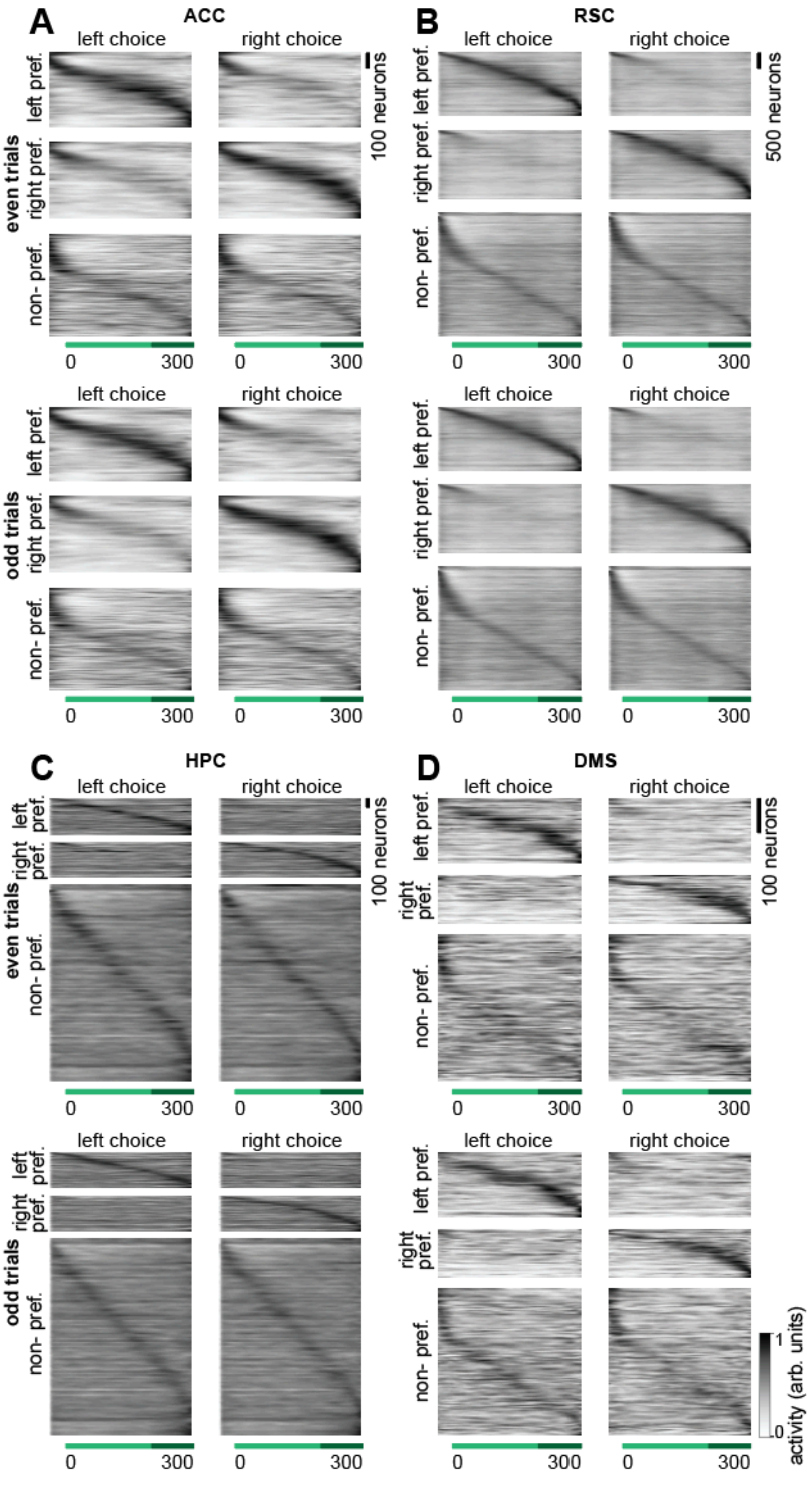

Supplementary Figure 1. Choice-selective sequences across brain regions during the accumulating towers task appear similar on even and odd trials. (A) Each row shows the

peak-normalized averaged firing rate (normalization based on the odd trials, with this odd-trial-based normalization then applied for cross-validation purposes to the neuronal responses on even trials) at each position in the maze of a neuron (see Methods) recorded during the accumulating towers task from ACC (n = 1720 neurons), averaged across even trials (top) or odd trials (bottom) when the animal turned left (left choice, left column) or right (right choice, right column). Neurons were divided based on their choice-selectivity (see Methods) and ordered based on the position of peak activity. **(B-D)** Same as (A) but for RSC (n = 8579 neurons; B), HPC (n = 3144 neurons; C), and DMS (n = 804 neurons; D).

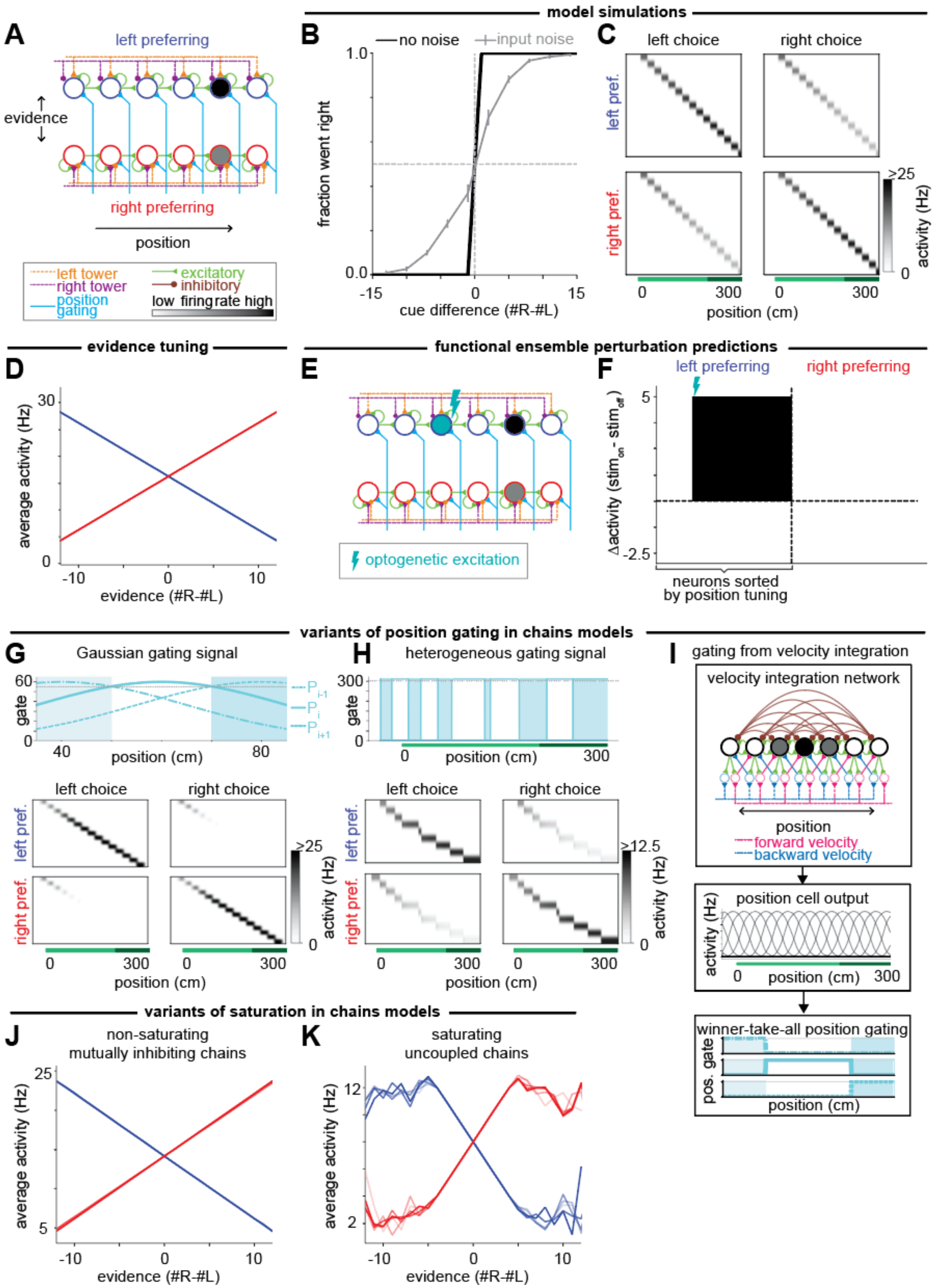

Supplementary Figure 2. Variants of competing chains models of evidence accumulation through sequences. (A) Schematic of neural circuit architecture for the uncoupled competing chains model,

showing excitatory (green) connections between neurons (circles) as well as inputs to the circuit from the external left (orange) and right (purple) towers and a position gating signal (cyan). **(B)** Psychometric data for model simulated trials describing how often the amplitude of the final neuron in the left chain was greater than that of the final neuron in the right chain for cases in which the model was simulated with (gray) and without (black) noise in the input. Error bars show s.e.m. **(C)** Each row shows the non-normalized amplitude of a model neuron at each position in the maze, averaged across simulated trials without input noise when the greater final amplitude was in the left (left choice, left column) or right (right choice, right column) chain. Neurons were divided based on their choice-selectivity (see Methods) and ordered based on the position of peak activity. **(D)** Tuning curves of individual neurons to evidence, defined by the average activity for different evidence levels at the neuron's peak position, for left-preferring (blue) and right-preferring (red) neurons. **(E)** Schematic of a single neuron perturbation experiment in which optogenetic excitation is applied to a single neuron in the left chain. **(F)** Simulated changes of the firing rates of all neurons in the absence of cues when a single cell (denoted with the laser as in E) is optogenetically stimulated. **(G)** Variant of the mutually inhibiting chains model with a Gaussian position gating signal (top). Neuronal responses (bottom) are as in panel C. **(H)** Same as G but for a square position gating signal of heterogeneous width. **(I)** Schematic of a velocity integration method for generating square position-gating signals, where velocity is integrated through a bump attractor to produce cells representing different preferred positions. A winner-take-all network takes these position cells as input to produce a square position gate. **(J)** As in D, but for a parameterization of the mutually inhibiting chains model (see Methods) which does not saturate within the observed evidence range. **(K)** As in D, but for a variant of the uncoupled competing chains model where an upper bound for saturation is enforced.

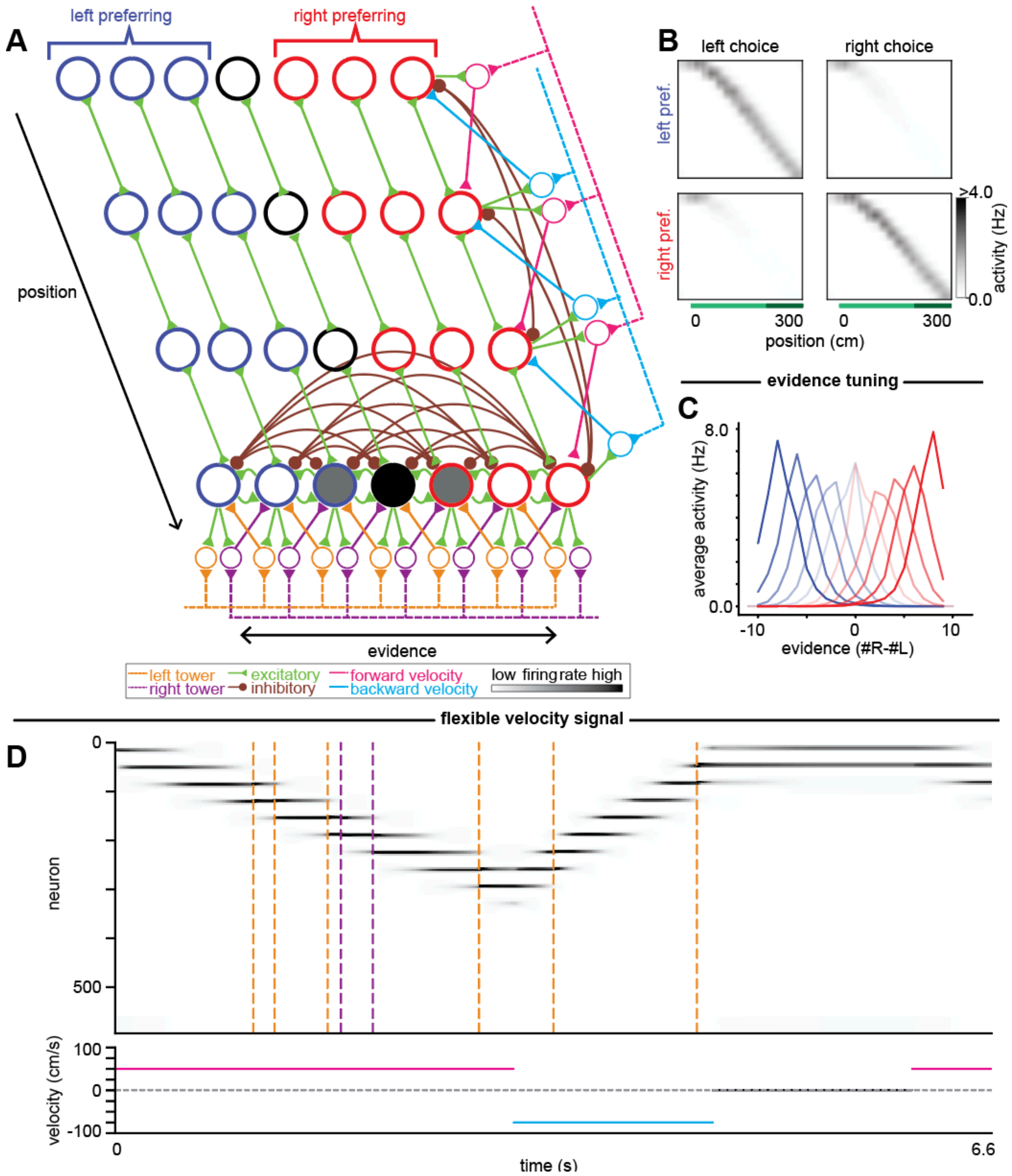

Supplementary Figure 3. Planar bump attractor that jointly accumulates evidence and position through sequences. (A) Schematic of the neural circuit architecture for the planar bump attractor. Each row represents neurons (circles) that respond at a given position. Within a row, each neuron represents a different evidence level, ranging from left-most to right-most. Blue: left-preferring neurons; red: right-preferring neurons. As in the position-gated bump attractor, the bottom row of neurons illustrates the connectivity within any given row and the cue-related inputs: local excitatory (green) and broader inhibitory (brown) connections between neurons as well as external inputs to the circuit from the left

(orange lines) and right (purple lines) towers via the corresponding shifter neurons (orange and purple circles). The rightmost column illustrates the velocity-related inputs and connectivity within any given column: local excitatory and broader inhibitory connections between neurons at different positions as well as either forward-direction (pink) or backward-direction (cyan) external velocity inputs via corresponding position-shifter neurons (pink and cyan circles). **(B)** Each row shows the non-normalized firing rate of a model neuron at each position in the maze, averaged across simulated trials without input noise when the greater final amplitude was in the left (left choice, left column) or right (right choice, right column) chain. Neurons were divided based on their choice-selectivity (see Methods) and ordered based on the position of peak activity. **(C)** Tuning curves of a subset of individual neurons to evidence, calculated at the position for which the neurons have peak activity, for left-preferring (blues) and right-preferring (reds) neurons. **(D)** Example simulation of the planar bump attractor for a hypothetical trial in which the animal can move both forwards and backwards. Top: Raster of activity of individual model neurons across different positions in the trial. Incoming towers are indicated by the dashed orange (left cue) and purple (right cue) lines. Neurons are ordered first by position and then by evidence within a given position. Bottom: The velocity of the animal at different points of the trial, including forward (pink) and backward (cyan) movement.

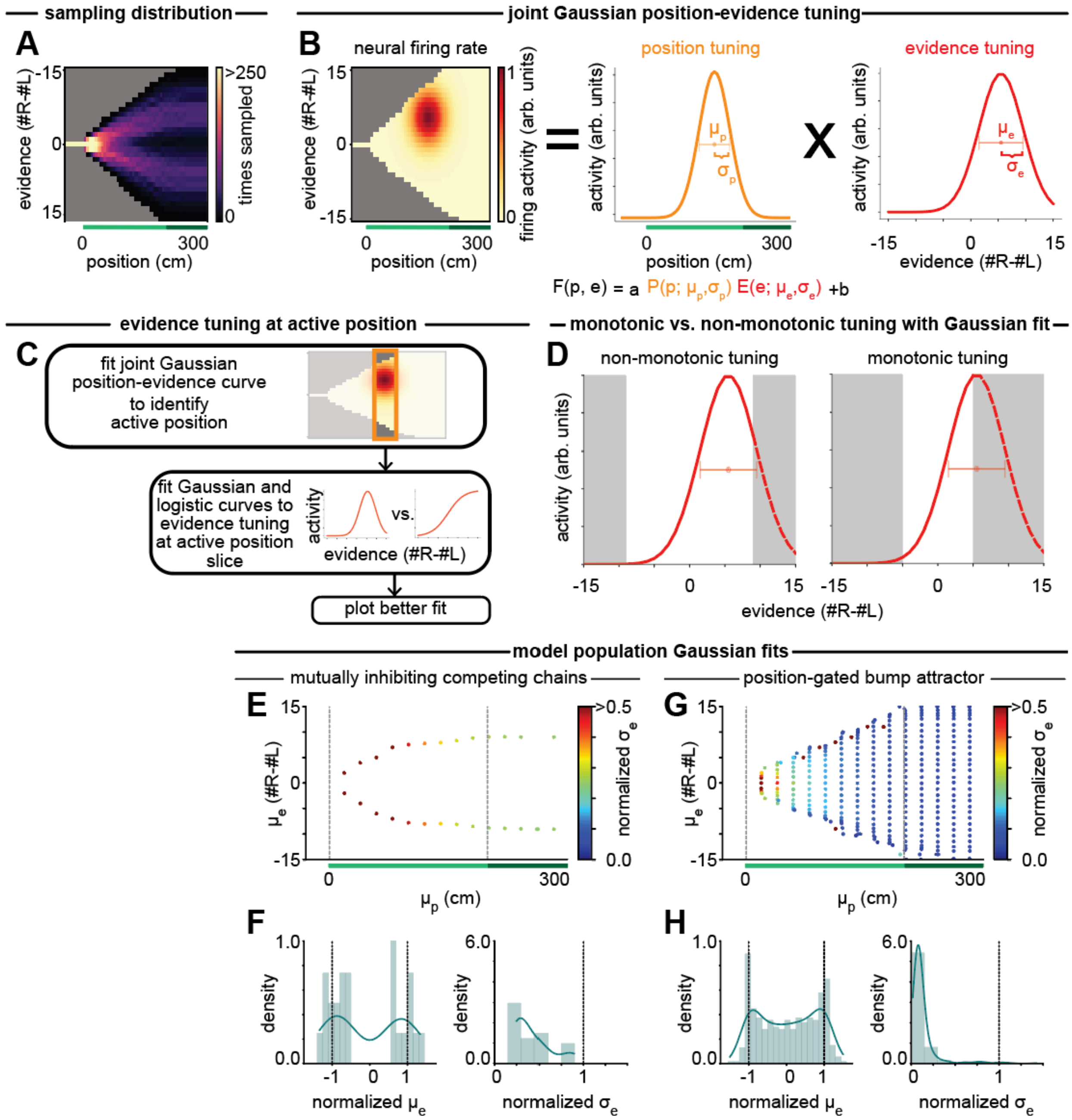

Supplementary Figure 4. Joint position-evidence tuning curve fitting procedure and Gaussian fits to the different models. (A) Number of times each position-evidence bin is sampled within the 1000 empirical trials used to simulate the models. The statistics of cue counts, distribution, and position used to generate trials make it such that at different positions, different evidence levels are not sampled uniformly. For example, neurons tuned to the beginning of the maze only see a small range of small evidence levels at the positions where they are most active; such a cell may not appear to respond to high levels of evidence because its position tuning is never active at these extreme evidence levels. **(B)** Fitting firing rates to a joint position-evidence Gaussian to identify μ_p, σ_p, μ_e, and σ_e. **(C)** Schematic of procedure for plotting evidence tuning curves by first identifying the active position region and then comparing the fit between a Gaussian and logistic curve. **(D)** Examples of how Gaussian fits can capture non-monotonic

(left) and monotonic (right) tuning to evidence, with the monotonicity of the fit curve dependent on the observed evidence range (gray regions indicate unobserved evidence levels). **(E)** Scatter plot showing the location of the fit mean position (μ_p) and fit mean evidence (μ_e) with color indicating the normalized fit evidence standard deviation (σ_e) (see Methods) of simulated neurons in the mutually inhibiting competing chains model. Dashed lines indicate the boundaries of the cue period. **(F)** Density plots of the normalized fit mean evidence (left) and the normalized fit evidence standard deviation (right) for the neurons shown in (E). **(G-H)** Same as for (E-F) but for simulated neurons from the position-gated bump attractor.

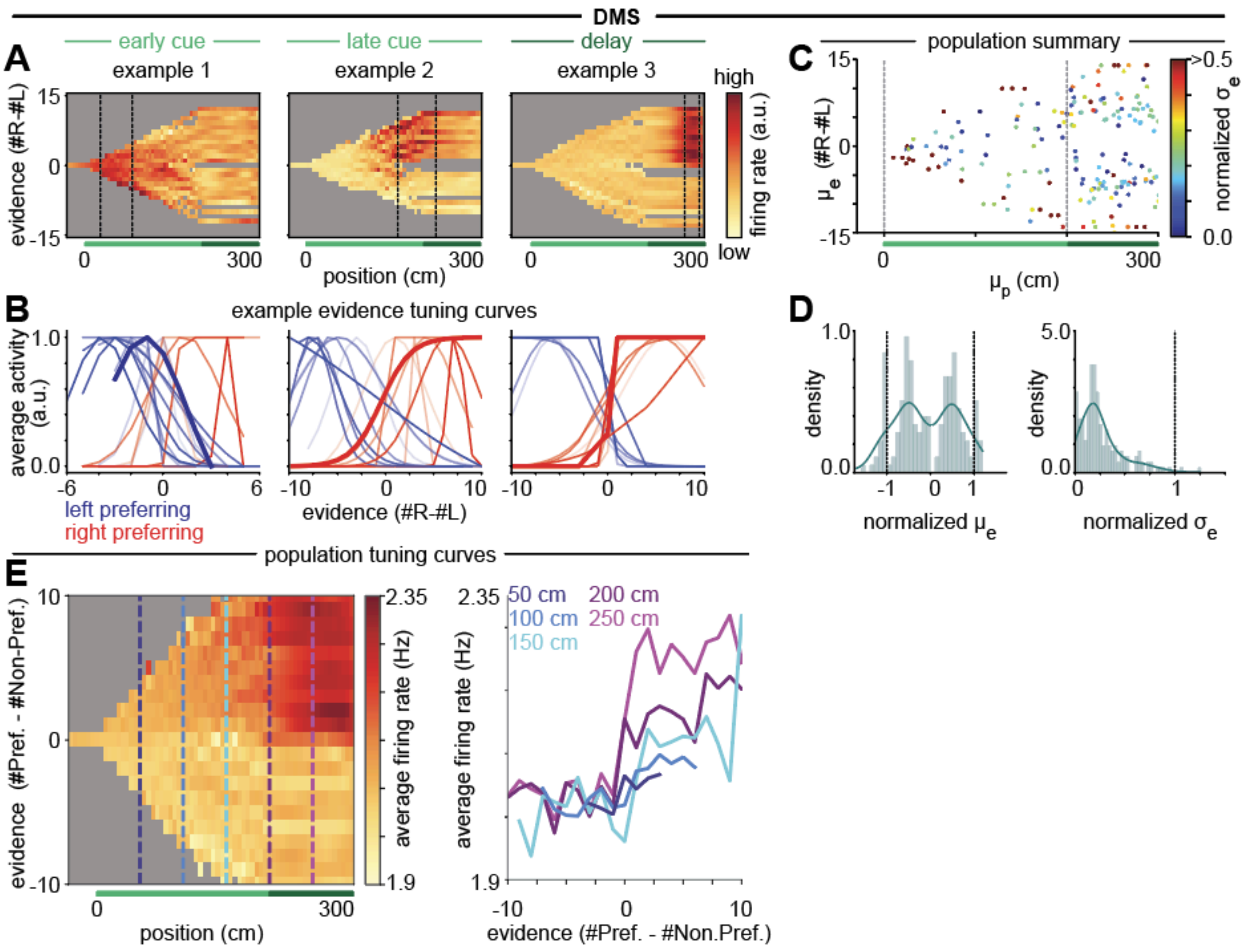

Supplementary Figure 5. DMS exhibits mainly monotonic evidence tuning dominated by neurons with position tuning in the delay region. (A) Heatmaps showing the average firing in position by evidence bins for example individual neurons in DMS with mean position in the early cue (left), late cue (middle), or delay (right) region of the maze. Gray bins denote regions for which there were fewer than 2 samples during the session. **(B)** Example DMS evidence tuning curves fit to the region of the neuron's peak activity (see Methods) for a collection of neurons with mean position tuning in the early cue (left), late cue (middle), or delay (right) region of the maze. Red coloring indicates neurons classified as right-preferring, and blue indicates left-preferring. Bold lines correspond to the examples in (A), for which the neuron's region of peak activity is the region between the dashed vertical lines. **(C)** Scatter plot showing the location of the fit mean position (μ_p) and fit mean evidence (μ_e) with color indicating the normalized fit evidence standard deviation (σ_e) (see Methods) of the 80% of neurons recorded in DMS with the best fit between the neural data and the model predictions. Dashed lines indicate the boundaries of the cue period. **(D)** Density plots showing the normalized fit mean evidence (left) and the normalized fit evidence standard deviation (right) for the neurons shown in (C). **(E)** Left: Heatmap showing average firing rates across all evidence-tuned neurons in bins of position by preferred-evidence. Gray indicates bins that were not sampled more than twice on at least 10% of sessions. Right: Cross-sections of the heatmap at points in the cue period (50 cm, dark blue; 100 cm, medium blue; 150 cm, cyan) and delay period (200 cm, purple; 250 cm, magenta), showing average firing rate across evidence-tuned neurons as a function of preferred evidence.

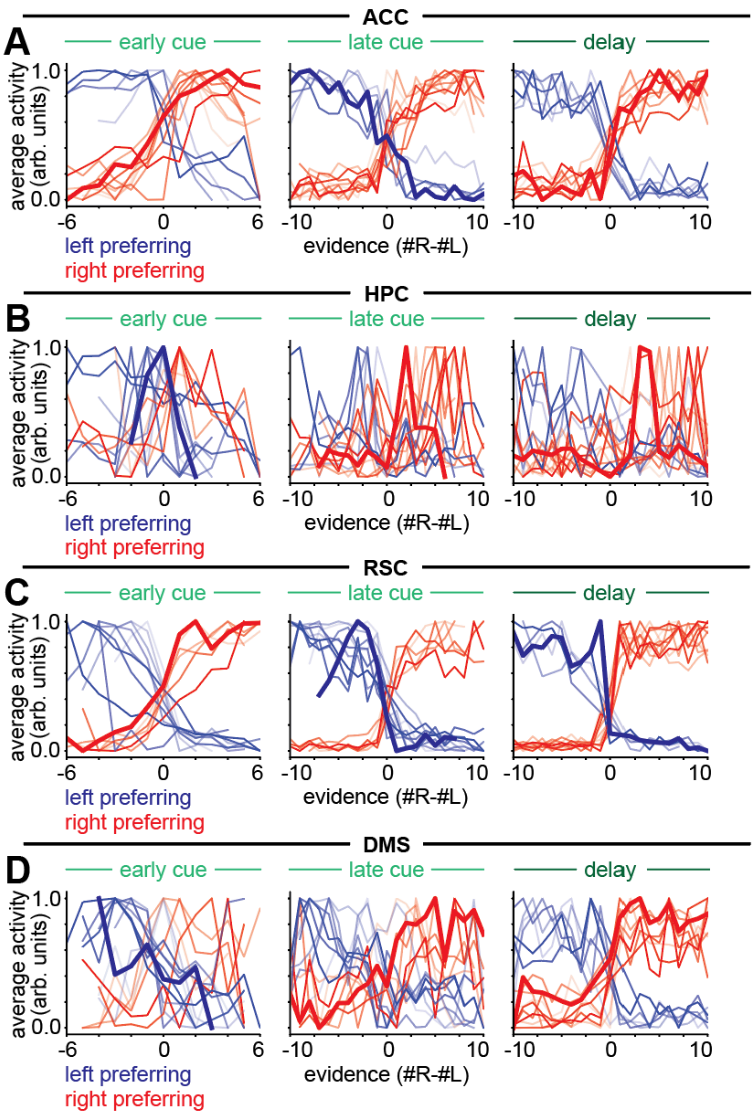

Supplementary Figure 6. Raw tuning curves for the four recorded brain regions. (A) Average activity of example ACC neurons at different evidence levels in the region of the neuron's peak activity (see Methods) for a collection of neurons with mean position tuning in the early cue (left), late cue (middle), or delay (right) region of the maze. Red coloring indicates neurons classified as right preferring, and blue indicates left preferring. Bold lines correspond to the examples plotted in Figure 5. **(B)** Same as (A) but for HPC. **(C)** Same as (A) but for RSC. **(D)** Same as (A) but for DMS, with the examples from Supplementary Fig. 5.

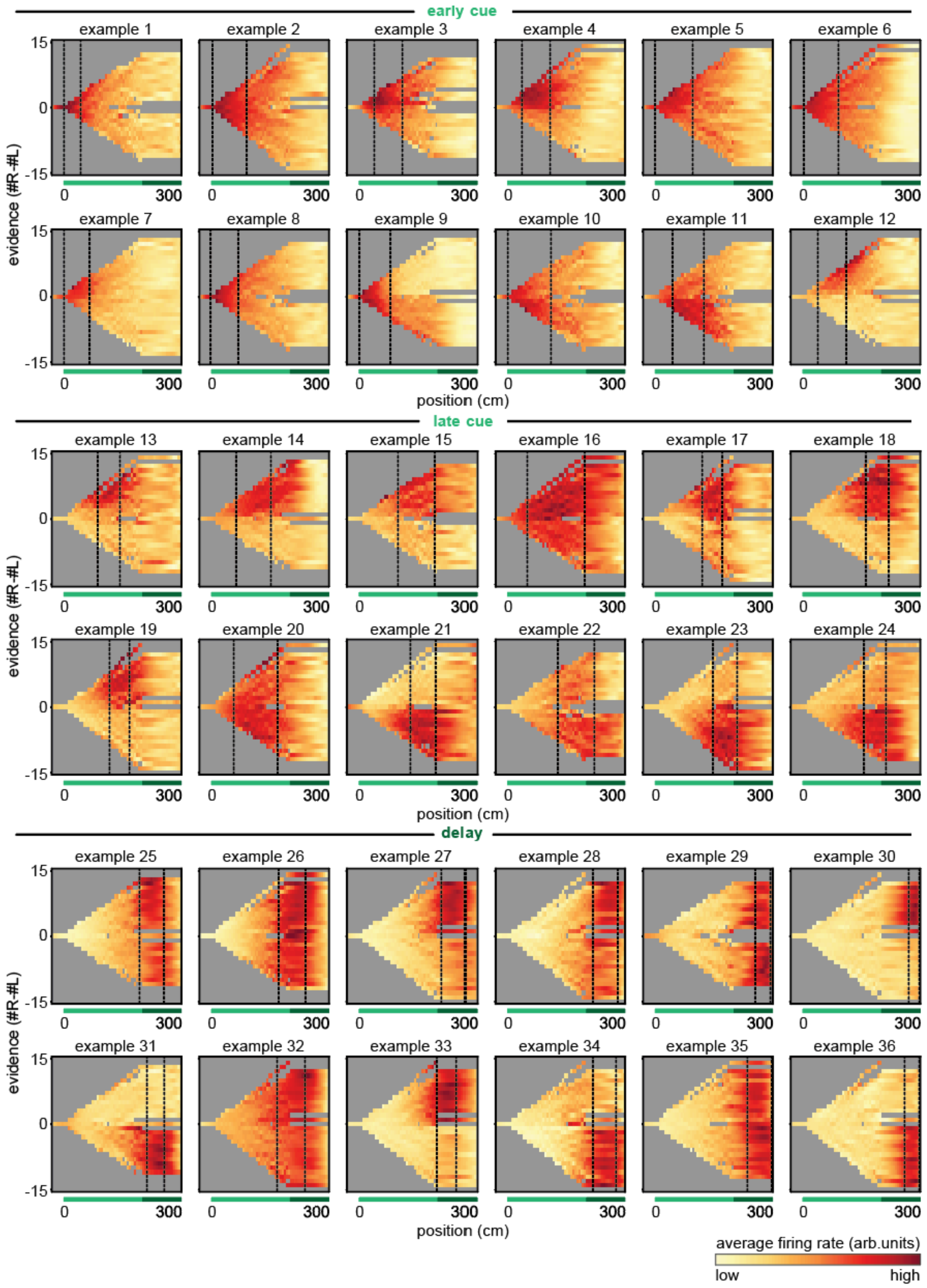

Supplementary Figure 7. Example evidence-tuned neurons in ACC. Heatmaps of average normalized firing activity within position-by-evidence bins of 36 example individual neurons from ACC with significant

evidence tuning (see Methods). Gray denotes position-evidence bins where there were fewer than 2 observations. A representative selection of neurons was made from neurons with mean position tuning in the early cue (0-100 cm), late cue (100-200 cm), and delay (200-300 cm) regions of the maze.

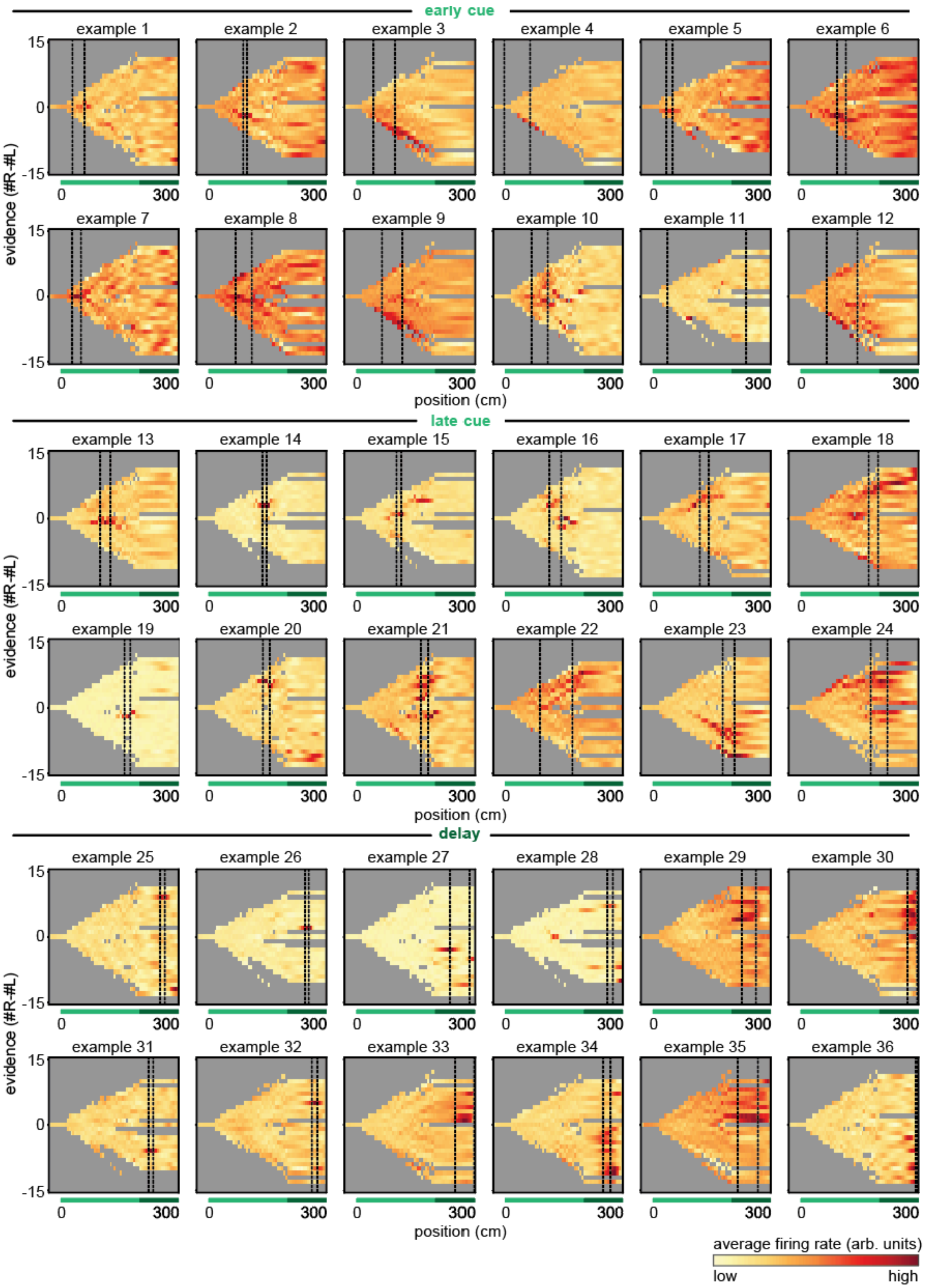

Supplementary Figure 8. Example evidence-tuned neurons in HPC. Same as in Supplementary Fig. 7 but for neurons from HPC.

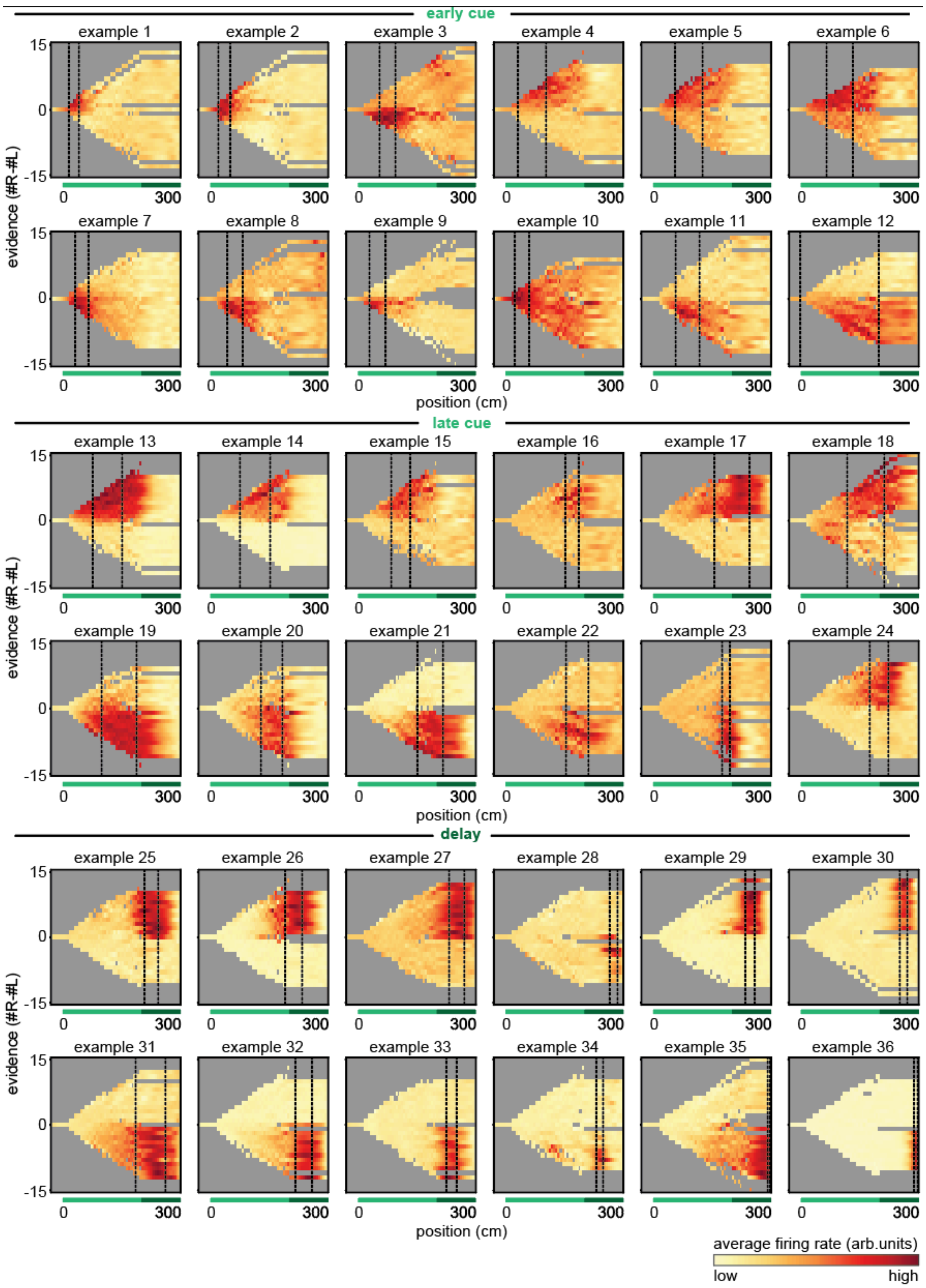

Supplementary Figure 9. Example evidence-tuned neurons in RSC. Same as in Supplementary Fig. 7 but for neurons from RSC.

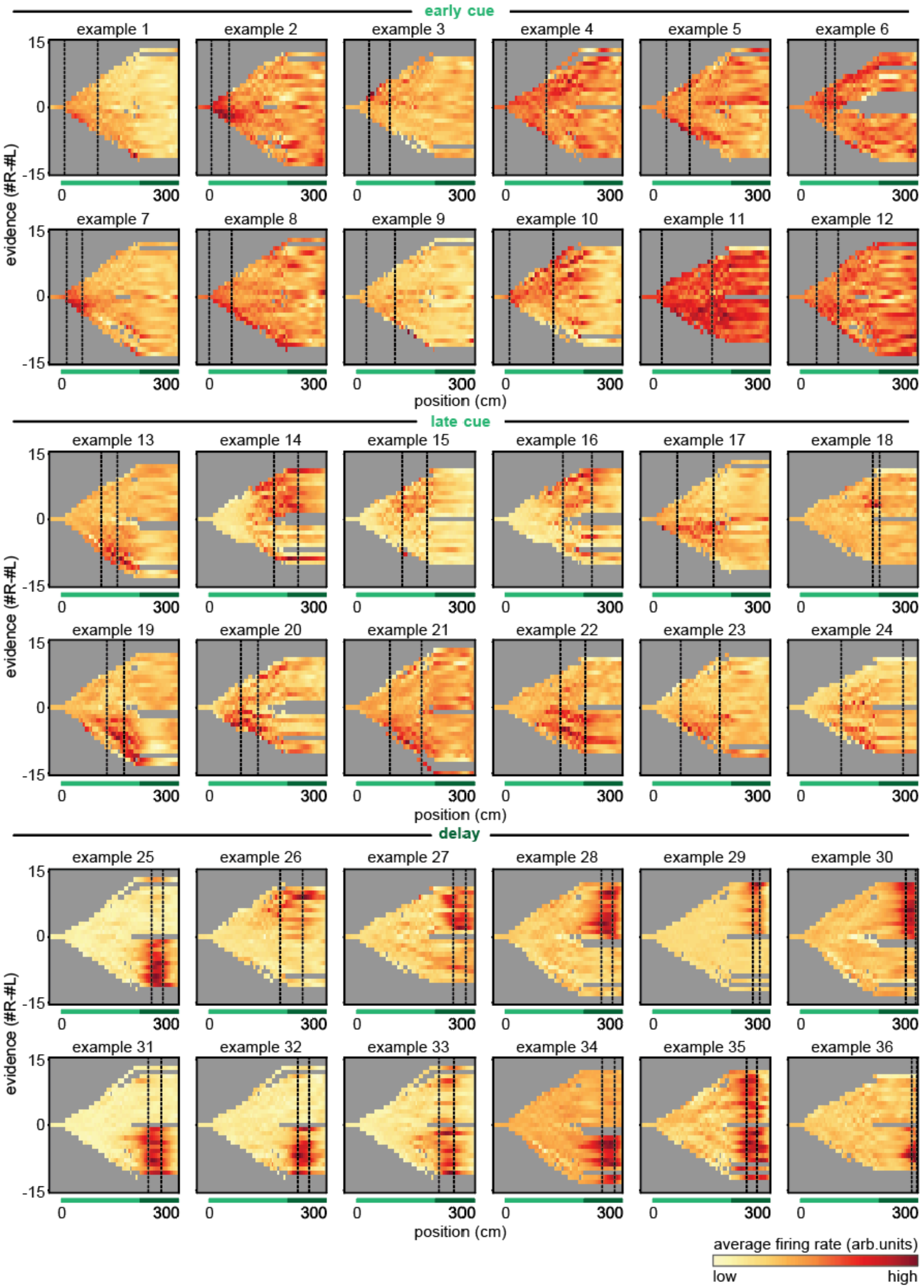

Supplementary Figure 10. Example evidence-tuned neurons in DMS. Same as in Supplementary Fig. 7 but for neurons from DMS.

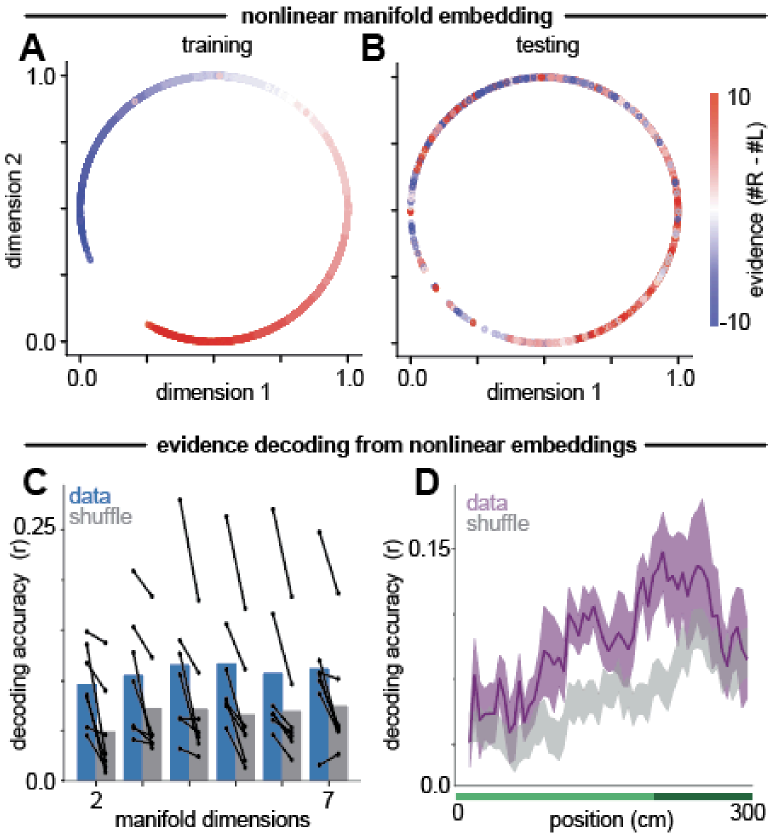

Supplementary Figure 11. Decoding evidence from HPC populations. (A) CEBRA embeddings uncover a nonlinear mapping that smoothly captures evidence levels (indicated by the color of the points) in the training set. **(B)** Same as in (A) but for the test set. **(C)** A k-nearest neighbors decoder can decode evidence from the CEBRA embedding (average performance measured by Pearson's correlation coefficient (r) between the actual and decoded evidence, blue) above a sign-of-evidence matched shuffle (gray). Lines indicate performance on individual sessions, compared to the corresponding shuffle. **(D)** Average decoding performance across different positions in the maze compared to a sign-of-evidence matched shuffle (gray) for 2-dimensional CEBRA embeddings. Error bars indicate s.e.m. across 7 sessions.

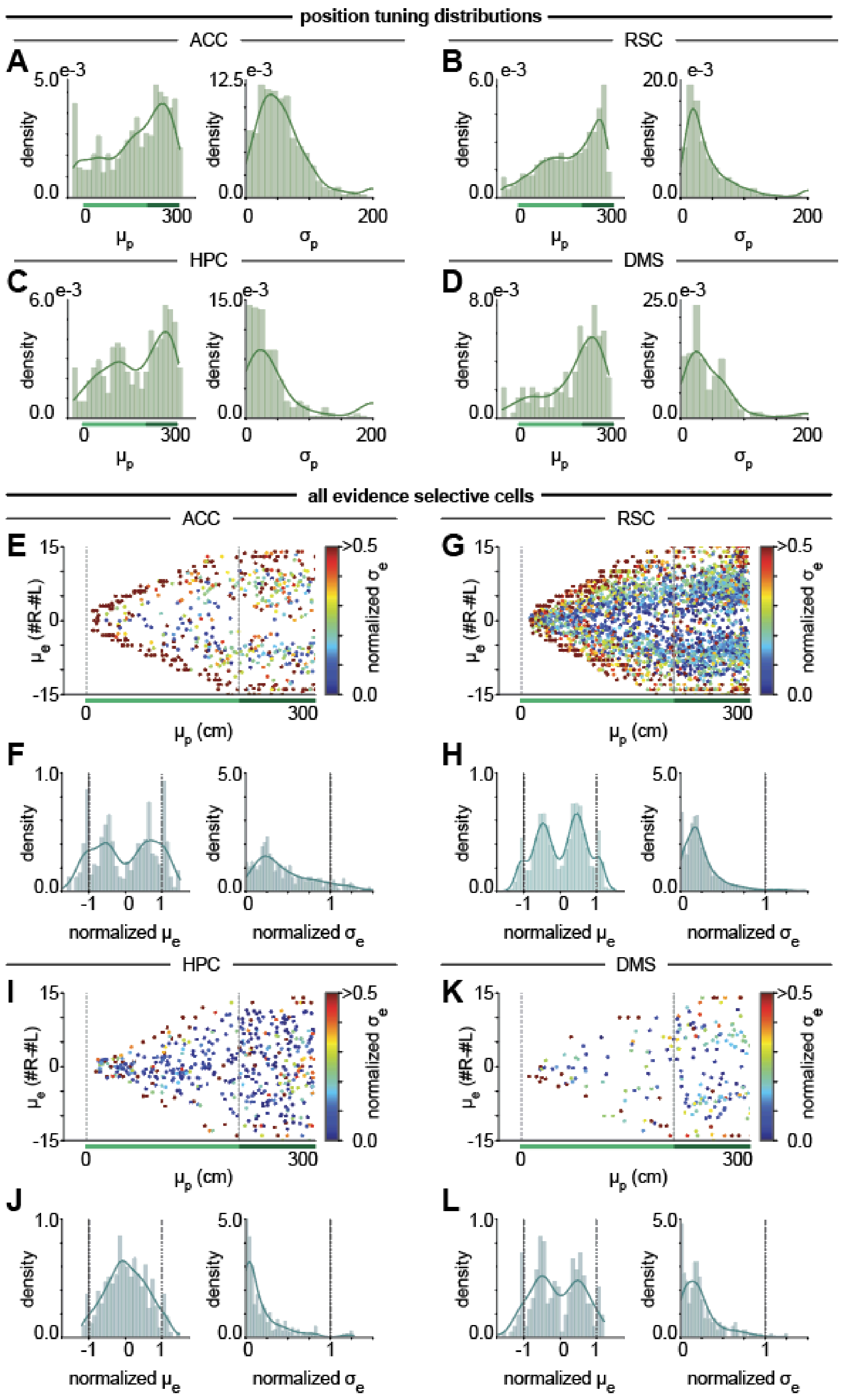

Supplementary Figure 12. Supplementary distribution plots. (A) Left: Distribution of μ_p for the neurons in ACC plotted in Figure 5. Right: Distribution of σ_p for the same neurons. **(B-D)** Same as (A) but

for neurons in RSC (B), HPC (C), and DMS (D), where DMS neurons are plotted in Supplementary Fig. 5. **(E)** Scatter of fit position mean vs. fit evidence mean colored by normalized σ_e for all significantly evidence-tuned neurons in ACC. **(F)** Left: Distribution of normalized μ_e for the neurons in E. Right: Distribution of normalized σ_e for the neurons in E. **(G-H)** Same as E-F but for RSC. Note that, by comparison to Figure 5, the narrowly tuned neurons in RSC (G, deep blue) tend to have poorer fits. **(I-J)** Same as E-F but for HPC. **(K-L)** Same as E-F but for DMS.

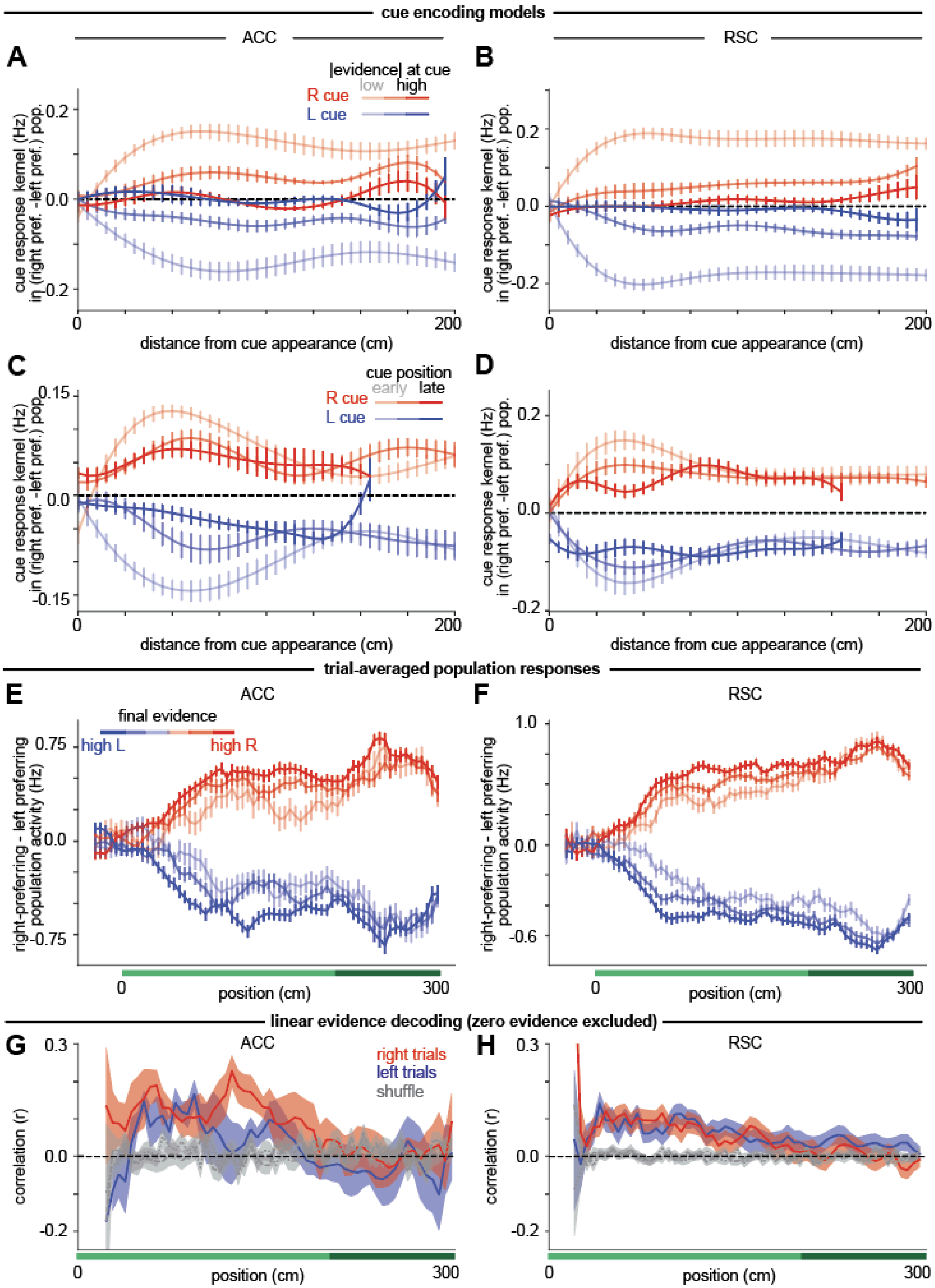

Supplementary Figure 13. Single trial signatures of graded evidence accumulation. (A) Change in the difference in activity between the right and left populations in ACC following a left cue (blue) or right

cue (red) when the current absolute value of evidence is low (light colors) to high (dark colors), where low is defined by $|e| \leq 1$, medium by $2 \leq |e| \leq 4$, or high by $|e| \geq 5$. (Average $r^2 = 0.29$ across 7 sessions.) **(B)** Same as (A) but for RSC. (Average $r^2 = 0.21$ across 41sessions.) **(C)** Change in the difference in activity between the right and left populations in ACC following a left cue (blue) or right cue (red) when the cue appears in the early (light colors) to late cue region (dark colors). (Average $r^2 = 0.27$ across sessions.) **(D)** Same as (C) but for RSC. (Average $r^2 = 0.20$ across sessions.) **(E)** For active cells in ACC with significant evidence coefficient in the single cell encoding analysis (see Methods), trial-averages of the difference between the mean activities of the left and right populations for trials with different final evidence levels (indicated by the color bar). **(F)** Same as E but for RSC. **(G)** At each position, the cross-validated correlation between actual evidence and predicted evidence from a linear population decoder in ACC on correct right evidence ($e > 0$) trials (red) and left evidence ($e < 0$) trials (blue), compared to shuffle (gray). Error bars indicate s.e.m. across sessions. **(H)** Same as (G) but for RSC.

SUPPLEMENTARY TEXT

In the following, we provide the mathematical analysis of our two classes of models. We show explicitly that, for the parameter conditions derived below, the models achieve the two fundamental operations of (i) evidence accumulation within a position and (ii) transfer of information between positions. We begin with an analysis of the competing chains models, for both the uncoupled competing chains models (Supplementary Fig. 2) and the mutually inhibiting competing chains models (Fig. 3), and conclude with the analysis of the position-gated bump attractor (Fig. 4) and planar bump attractor (Supplementary Fig. 3).

COMPETING CHAINS MODELS

The competing chains models represent evidence in the difference of activity of two chains of neurons. In principle, these chains could be completely independent, with each chain integrating the cues only to its respective side, but this would result in chains that only increase in amplitude across the trial, inconsistent with observed data. Instead, we consider architectures with ipsilateral excitation and contralateral inhibition, where the contralateral inhibition comes either from external inputs or from the other chain. Specifically, we consider: (i) a model in which the chains are uncoupled but towers appearing to the ipsilateral side are excitatory and towers appearing to the contralateral side are inhibitory (the uncoupled competing chains model, Supplementary Fig. 2A) and (ii) a model in which each chain receives input only from the ipsilateral side and competes with the other chain through mutual inhibition within the same position (the mutually inhibiting competing chains model, Fig. 3A).

Basic Features of Competing Chains Models

The neurons in each model can be represented by the same basic set of differential equations, where the firing rate of the ith neuron in the left chain, $r_{i,L}$, evolves according to

$$\frac{dr_{i,L}}{dt} = -ar_{i,L} + [br_{i,L} + cr_{i-1,L} - er_{i,R} + f\mathbf{1}_{left}(t) - g\mathbf{1}_{right}(t) + P_i(t) - T]^+ \quad \text{(S1)}$$

and similarly for the firing rate of neurons in the right chain. The various terms on the right side of the equation are described in the main text (see text below Eq. (1)), although here we add a term $g\mathbf{1}_{right}(t)$ that allows for inhibitory external inputs from cues on the contralateral side. The external cue inputs are given by $\mathbf{1}_{left}(t)$ for the left inputs and $\mathbf{1}_{right}(t)$ for the right inputs, which take the form

$$\mathbf{1}_{\text{left}}(t) = \begin{cases} 1 & \min_{\ell \in L} |t - \ell| < 0.5 \\ 0 & \text{otherwise} \end{cases}$$

and

$$\mathbf{1}_{\text{right}}(t) = \begin{cases} 1 & \min_{R \in \mathcal{R}} |t - R| < 0.5 \\ 0 & \text{otherwise} \end{cases}$$

where L is the set of times of left cues and $\mathcal{R}$ is the set of times of right cues.

The position signal is given by

$$P_i(t) = \begin{cases} T + X(t) & i \leq \frac{p(t)}{P_0} < (i+1) \\ 0 & \text{otherwise} \end{cases},$$

where $X(t) \geq 0$ and $p(t)$ is the position of the animal along the maze, so that each neuron receives an active position signal for length P_0, and only one neuron in each chain receives an active signal at any time.

For both models, we require the chains to exhibit a number of properties in order to accurately accumulate evidence, so that at the end of a trial, the chain corresponding to the side with more cues will have greater amplitude. In particular, we look to achieve the following properties: (i) Each neuron is only active around a specific position, tiling space, with activity decaying away when the simulated animal leaves this position. (ii) External cue inputs are integrated linearly. (iii) In the absence of external cue inputs, when at a fixed position, the difference in neural activity of the two chains remains constant, without the sum growing unboundedly. (iv) In the absence of external cue inputs, the difference in amplitude of the two chains is preserved across positions. We analyze the necessary conditions on the model parameters to achieve each of these properties.

Uncoupled Competing Chains Model

For the case of the uncoupled competing chains model (Supplementary Fig. 2), we take $e = 0$, giving us

$$\frac{dr_{i,L}}{dt} = -ar_{i,L} + [br_{i,L} + cr_{i-1,L} + f\mathbf{1}_{left}(t) - g\mathbf{1}_{right}(t) + P_i(t) - T]^+ \quad \text{(S2)}.$$

We consider below the conditions needed for this model to achieve the desired properties.

Neurons Active Only Around a Specific Position

Activity only occurring around a specific position when the simulated animal is navigating down the maze is achieved through the position-gating mechanism. We set the threshold T of the neuron sufficiently high that the term in square brackets will only affect the firing rate of the neuron in the presence of the position gating input $P_i(t)$. Otherwise, neural activity decays exponentially. This leads to the transient, sequential responses.

External Cues Integrated Linearly

In order for each cue to be integrated linearly, first, each cue should be weighted equally in its contribution to the final activity of the chain. This is achieved by giving each cue the same form of input in $\mathbf{1}_{left}(t)$ and $\mathbf{1}_{right}(t)$.

Second, this signal must be fully integrated by the chain. Since the external cue input term resides in the square brackets, only the neurons with the active position gating signal can integrate this signal. Without loss of generality, we consider the left-side chain. For the left-side neuron with an active position gating signal,

$$\frac{dr_{i,L}}{dt} = -ar_{i,L} + [br_{i,L} + cr_{i-1,L} + f\mathbf{1}_{left}(t) - g\mathbf{1}_{right}(t) + X(t)]^+.$$

For this neuron to integrate its inputs, we need the term inside square brackets to be positive. We can see that this is guaranteed if $br_{i,L} + cr_{i-1,L}$ is greater than $-g$. We show below that the

difference in activity of the chains is preserved across positions in the absence of external cues when $b=c=a$, so that $r_{i,L}+r_{i-1,L} = C$ for some constant C. Thus, by initializing the activity at the start of the trial, $r_{0,L}$, to be sufficiently large that the activity of the lower firing rate chain does not drop below g/a, these inputs will always be integrated.

We note that the argument above does not require the cues to have the square pulse specified here. Rather, it only requires that each cue has the same form.

Difference in Activity Between Chains Remains Constant at a Fixed Position

When the animal maintains a fixed position, the activity of the neurons at the preceding position decay away, so that in the absence of external cue inputs, we have

$$\frac{dr_{i,L}}{dt} = -ar_{i,L} + [br_{i,L} + P_i(t) - T]^+ .$$

If we take $X(t)$=0, we have $P_i(t) = T$ at the active position, and thus

$$\frac{dr_{i,L}}{dt} = -ar_{i,L} + [br_{i,L}]^+ = -ar_{i,L} + br_{i,L} .$$

Thus, when $a = b$, each chain maintains constant activity, both preserving the difference between the chains and keeping the sum constant.

If instead $X(t) > 0$, this signal will be integrated, causing both chains to increase in amplitude. Such a signal could be designed to act as an urgency signal, but must be carefully crafted to prevent the activity in the chains from growing too rapidly.

Difference in Amplitude Preserved Across Positions

Preserving the amplitude of the chain across positions is a special case of preserving the difference across positions. To preserve amplitude across positions in the absence of cues, the next neuron in the chain must integrate the activity of the previous neuron at the same rate at which it decays. At the active position in the absence of external cue inputs, and assuming $X(t)$=0, we have

$$\frac{dr_{i,L}}{dt} = -ar_{i,L} + [ar_{i,L} + cr_{i-1,L}]^+$$

$$\frac{dr_{i,L}}{dt} = cr_{i-1,L}$$

while we also have

$$\frac{dr_{i-1,L}}{dt} = -ar_{i-1,L}$$

To preserve the total amplitude of activity,

$$-\frac{dr_{i-1,L}}{dt} = \frac{dr_{i,L}}{dt} ,$$

giving us the condition $a = c$.

We note that neuron i will only fully integrate the activity of the previous position in the infinite time limit. Due to the transient nature of the position-gating signal, which is active for the length of the position signal P_0 or equivalently for time P_0/v for an animal traveling at constant

velocity *v,* we have that the final amplitude of the ith neuron after the animal has traversed the length of the position gating signal will be

$$r_{i,L}(\frac{P_0}{v}) = \int_0^{\frac{P_0}{v}} ar_{i-1,L}dt = \int_0^{\frac{P_0}{v}} ar_{i-1,L}(0)e^{-at}dt = r_{i-1,L}(0)\left(1 - e^{-a\frac{P_0}{v}}\right).$$

For *a* and P_0 sufficiently large or *v* sufficiently small, the difference in amplitude between positions will be exponentially small.

Previous analysis of mouse running speed versus performance has shown little correlation within a session and a positive correlation on average across all sessions (see Supplementary Figure 7 in Pinto et al. (2018)[1]), suggesting that mice do not run at such a fast speed that information loss is problematic. Alternatively, additional connections could support the transfer of information from longer distances in the chain, increasing robustness to information loss.

Parameterization of the Uncoupled Competing Chains Model

In our simulations, in addition to taking $X(t)$ = 0 Hz/s, we set a = 50 s^{-1}, b = 50 s^{-1}, c = 50 s^{-1}, f = 50 Hz/s, g = 50 Hz/s, P_0 = 20 cm, and T = 15000 Hz/s. Neurons $r_{0,L}$ and $r_{0,R}$ are initialized to 16.25 Hz activity level. We note that the neurons must be initialized to a sufficiently high non-zero activity level such that an external input to the contralateral side can be negatively integrated into the activity.

The Uncoupled Competing Chains Model with Saturation

In Supplementary Figure 2K, we present a version of the uncoupled chains model that has saturation in its firing. This was enforced by placing an upper bound on its firing rate. Note that because the dynamics are governed by the sum of an exponential decay and a non-negatively thresholded term, the firing rate of the neuron will be lower bounded at zero. To enforce the upper bound, we take

$$z(t) = -ar_{i,L} + [br_{i,L} + cr_{i-1,L} + f\mathbf{1}_{left}(t) - g\mathbf{1}_{right}(t) + P_i(t) - T]^+$$

and

$$\frac{dr_{i,L}}{dt} = \begin{cases} z(t) & r_{i,L} \leq r_{\max} \\ \min(z(t), 0) & \text{otherwise} \end{cases},$$

where r_{max} is the upper bound on the firing rate, which we set to 13 Hz in our simulations.

Mutually Inhibiting Competing Chains Model

In this model, the chains compete through mutual inhibition, rather than receiving opposing inputs. For the mutually inhibiting competing chains model, we take g = 0 in Eq. (S1), giving

$$\frac{dr_{i,L}}{dt} = -ar_{i,L} + [br_{i,L} + cr_{i-1,L} - er_{i,R} + f\mathbf{1}_{left}(t) + P_i(t) - T]^+.$$

We next present the necessary conditions for this model to achieve the desired properties.

Neurons Active Only Around a Specific Position

Activity only occurring around a specific position when the simulated animal is navigating down the maze is achieved through the same position gating mechanism as before, with T much larger than the other terms in square brackets so that the neuron will only be active when the position gate $P_i(t)$ is active.

External Cues Integrated Linearly

Each cue should be weighted equally in its contribution to the final activity of the chain. This is achieved by giving each cue the same form of input in $\mathbf{1}_{left}(t)$ and $\mathbf{1}_{right}(t)$. Moreover, the weights of mutual inhibition and self-excitation must be tuned such that the system of equations is a perfect integrator when the term in square brackets is above threshold. The position gating ensures that this can be true for at most two neurons, both at the same position. Specifically, when the term in square brackets is above threshold, we have

$$\frac{d}{dt}\begin{pmatrix} r_{i,L} \\ r_{i,R} \end{pmatrix} = \begin{pmatrix} b-a & -e \\ -e & b-a \end{pmatrix}\begin{pmatrix} r_{i,L} \\ r_{i,R} \end{pmatrix} + \begin{pmatrix} cr_{i-1,L} + f\mathbf{1}_{left}(t) + X(t) \\ cr_{i-1,R} + f\mathbf{1}_{right}(t) + X(t) \end{pmatrix},$$

which has eigenvalues (b-a-e) for the common mode eigenvector, (1, 1), and (b-a+e) for the difference mode eigenvector, (1, -1). For a perfect integrator that accumulates evidence in the difference of firing between the two chains, we require that the eigenvalue corresponding to the difference mode eigenvector is zero, giving $e = a - b$. Due to the mutual inhibition architecture, the eigenvalue corresponding to the common mode (b-a-e) is negative (corresponding to decay). We note that, for the assumption that both neurons are above threshold to be true, we require $X(t) + br_{i,L} + cr_{i-1,L} - er_{i,R} > 0$ and $X(t) + br_{i,R} + cr_{i-1,R} - er_{i,L} > 0$. This can be achieved by setting $X(t) = I_{ext}$ for some sufficiently large constant I_{ext}, and integration will only be perfect in the range over which this is true. This common external input to both chains serves as a background about which integration occurs at each position in the chain.

Difference in Activity Between Chains Remains Constant at a Fixed Position

Holding the difference in activity between the neurons in the chain constant at a constant position is a natural consequence of having a perfect integrator for the difference mode eigenvector. Furthermore, because the common mode eigenvector is associated with a negative (decay-associated) eigenvalue, the sum of activity will not grow uncontrollably.

Difference in Amplitude Preserved Across Positions

To appropriately represent the difference in accumulated evidence between the chains, we require the difference $\Delta_i = r_{i,L} - r_{i,R}$ between the chains to be preserved between positions. This difference evolves over time according to

$$\frac{d\Delta_i}{dt} = -ar_{i,L} + br_{i,L} + cr_{i-1,L} - er_{i,R} - (-ar_{i,R} + br_{i,R} + cr_{i-1,R} - er_{i,L})$$

$$\frac{d\Delta_i}{dt} = (-a + b + e)r_{i,L} + (a - b - e)r_{i,R} + c(r_{i-1,L} - r_{i-1,R})$$

$$\frac{d\Delta_i}{dt} = (-a+b+e)\Delta_i + c\Delta_{i-1}$$

From our conditions on perfect integration, we have (-*a* + *b* + *e*) = 0, giving us

$$\frac{d\Delta_i}{dt} = c\Delta_{i-1}.$$

We also have that

$$\frac{d\Delta_{i-1}}{dt} = -ar_{i-1,L} + ar_{i-1,R} = -a\Delta_{i-1}$$

For the total difference in activity of the chains to be preserved, we require

$$-\frac{d\Delta_{i-1}}{dt} = \frac{d\Delta_i}{dt},$$

giving the condition $a = c$.

As in the case of the uncoupled chains model, due to the finite time of integration of the signal from the previous neuron in the chain, we have that

$$\Delta_i(\frac{P_0}{v}) = \Delta_{i-1}(0)\left(1 - e^{\frac{-aP_0}{v}}\right)$$

so that the difference between positions will be exponentially small for a and P_0 sufficiently large or v sufficiently small.

POSITION-GATED BUMP ATTRACTOR

Bump attractor based models have historically been used to model heading direction[2–13] and path integration[14–16]. In this work, we modified the bump attractor model to be position-gated, so that within each position, there is a bump attractor that integrates visual cues. To accurately accumulate evidence, we require this model to exhibit several properties. Specifically, we show below that our model has the following properties: (i) Each neuron is only active around a specific position, tiling space, with the exact neurons that are active determined by the value of accumulated evidence. When the simulated animal leaves this position, activity decays away. (ii) In the absence of external inputs, when at a fixed position, the bump remains fixed. (iii) External cue inputs cause the bump to shift in the direction of the input, with the magnitude of the shift independent of the current bump location. (iv) In the absence of external cue inputs, the location of the bump along the evidence axis is maintained across positions.

Recall from Eq. (2) that our position-gated bump attractor dynamics for a neuron at position *i* and evidence *j* are governed by

$$\frac{dr_{i,j}}{dt} = -ar_{i,j} + F\left(\sum_k W_{j,k} r_{i,k} + br_{i-1,j} + c\left(I_{i,j+1,L}(t) + I_{i,j-1,R}(t)\right) + P_i(t) - T\right) \quad \text{(S3)}$$

where

$$I_{i,j,L} = r_{i,j}\mathbf{1}_{\text{left}}(t), \quad \text{(S4)}$$

$$I_{i,j,R} = r_{i,j}\mathbf{1}_{\text{right}}(t), \quad \text{(S5)}$$

and where

$$\mathbf{1}_{\text{left}}(t) = \begin{cases} 1 & \min_{\ell \in L} |t - \ell| < 0.5 \\ 0 & \text{otherwise} \end{cases}$$

and

$$\mathbf{1}_{\text{right}}(t) = \begin{cases} 1 & \min_{R\in\mathcal{R}} |t - R| < 0.5 \\ 0 & \text{otherwise} \end{cases}$$

where L is the set of times of left cues and $\mathcal{R}$ is the set of times of right cues. The various terms on the right side of the equation are described in the main text (see text below Eq. (2)). We use a sigmoidal nonlinearity

$$F(x) = \frac{q(1 + \tanh(\gamma x))}{2}$$

with $q > 0$ and synaptic connection strengths governed by

$$W_{j,k} = \omega_0(\cos(\theta_j - \theta_k) + \omega_1).$$

Although formally this synaptic connection matrix is circularly symmetric, we assume (and appropriately define parameters) such that we are working in a regime where the number of neurons is much larger than the space of observed evidence levels (see Methods). Thus, we do not encounter any effects from the circular boundary conditions. Alternatively, the tuning can be accomplished by adjusting the weights appropriately near the extremes of evidence[17,18]. Here, we use circular boundary conditions because this mathematical simplification facilitates the mathematical demonstration of the tuning conditions for the network provided below.

As in the competing chains models, the position signal is given by

$$P_i(t) = \begin{cases} T + X(t) & i \leq \frac{p(t)}{P_0} < (i+1) \\ 0 & \text{otherwise} \end{cases}$$

for $p(t)$ the position of the animal along the maze at time t.

We consider below the conditions needed for this model to achieve the desired properties.

Neurons Active Only Around a Specific Position

Activity only occurring around a specific position when the simulated animal is navigating down the maze is achieved by the nonlinearity F saturating at 0 for large negative input. When the position gate $P_i(t) \geq T$, the bump attractor becomes active in layer i. For all other layers, for T sufficiently large, the activities of all neurons decay temporally with time constant $1/a$, leading to sequential, transient responses.

Bump is Stable Within a Position in the Absence of Input

At the active position, $P_i(t) \geq T$ and we assume that the position-gating signal is constant, with $X(t) = X_0$. Assume that the activity at position i-1 has decayed to be sufficiently small, and that there are no external cues. A stationary solution will satisfy

$$0 = -ar_{i,j} + F\left(\sum_k W_{j,k} r_{i,k} + X_0\right).$$

Since the entries of W depend only on $|j - k|$, any rigid translation of this stationary solution will also be stationary. Furthermore, these weights also guarantee that a stationary solution will be

symmetric. A stationary solution centered at evidence *j* will correspond to a solution of the equations

$$ar_{i,j} = F\left(\sum_k W_{j,k} r_{i,k} + X_0\right).$$

For ease of analysis, we approximate the discrete sum above by an integral. This is done to facilitate the analysis below of moving the bump along the direction of the attractor and strictly would correspond to the limit of having sufficiently close together evidence levels that they form a continuum, parameterized by θ at position *i*, with corresponding firing rates at each evidence level given by $r(\theta)$. In this continuum limit, at any given position, we have

$$ar(\theta) = F\left(\int_{-\pi}^{\pi} w(\theta-\hat{\theta}) r(\hat{\theta}) d\hat{\theta} + X_0\right),$$

where

$$w(\theta-\hat{\theta}) = \omega_0(\cos(\theta-\hat{\theta}) + \omega_1).$$

By the symmetry arguments above, we have that if $r(\theta)$ is a stationary solution, $r(\theta+\delta)$ is also a stationary solution.

To examine the stability of this solution at the active position, we return to the original differential equation, in the continuum limit,

$$\frac{dr(\theta)}{dt} = -ar(\theta) + F\left(\int_{-\pi}^{\pi} w(\theta-\hat{\theta}) r(\hat{\theta}) d\hat{\theta} + X_0\right).$$

Taking the weighted integral of each side of the equation,

$$\int_{-\pi}^{\pi} w(\theta-\theta') \frac{dr(\theta')}{dt} d\theta' = \int_{-\pi}^{\pi} w(\theta-\theta') \left[-ar(\theta') + F\left(\int_{-\pi}^{\pi} w(\theta'-\hat{\theta}) r(\hat{\theta}) d\hat{\theta} + X_0\right)\right] d\theta'$$

and defining

$$y(\theta) = \int_{-\pi}^{\pi} w(\theta-\theta') r(\theta') d\theta' + X_0,$$

which can be interpreted as the total input current to the neuron whose tuning is centered at position θ, we have

$$\frac{dy(\theta)}{dt} = -ay(\theta) + \int_{-\pi}^{\pi} w(\theta-\theta') F(y(\theta')) d\theta' + aX_0.$$

With this transformation, our equation is now in the same form as in the standard proofs that this stationary solution is a neutrally stable attractor, rather than an unstable solution. We do not repeat the proof here, and instead refer the reader to Kishimoto and Amari (1979)[19].

We note that X_0 along with the synaptic connectivity determines the width of the bump. For a given level of synaptic connectivity, increasing X_0 will lift up the bump, making the super-threshold portion wider, and decreasing X_0 will lower the bump and make it narrower.

External Cues Cause a Shift in the Bump

We next show that inputs to the model cause the bump to shift and that the shift is linear in the number of cues. Inputs to the model in Eq. (S3) are given by Eqs. (S4) and (S5).

In the continuous framework, we have input to $r(\theta)$ of the form

$$r(\theta+\delta)\mathbf{1}_{\text{left}}(t) + r(\theta-\delta)\mathbf{1}_{\text{right}}$$

where δ is the magnitude of the asymmetric shift in the connections from the shifter neurons (orange and purple circles in Fig. 4A) to the evidence neurons.

We noted above that this model supports a bump of activity that is neutrally stable since all translations of this bump are also stationary. Neutral stability makes it such that inputs along the direction of the attractor cause a shift in the location of the bump, while inputs perpendicular to the attractor direction decay away without shifting the bump.

In the previous section, we showed that in the continuum limit, the attractor satisfies

$$ar(\theta) = F\left(\int_{-\pi}^{\pi} w(\theta-\hat{\theta})r(\hat{\theta})d\hat{\theta} + X_0\right).$$

Define **r***(θ) to be the solution (i.e., vector of steady state neuronal firing rates) that is centered at $\theta = 0$. We note that this should not be confused with the non-boldface $r^*(\theta)$ that occurs below, which we use to denote the firing rate of the single neuron that has preferred evidence level θ when the network is at its stationary solution. Without loss of generality, we consider inputs to the network when it is at the stationary solution **r***(θ), as inputs when the network is at other stationary solutions are equivalent, up to translation, by symmetry. The direction along the attractor is given by

$$\frac{d\mathbf{r}^*(\theta)}{d\theta} = \frac{1}{a}F'\left(\int_{-\pi}^{\pi} w(\theta-\hat{\theta})\mathbf{r}^*(\hat{\theta})d\hat{\theta} + X_0\right) \odot \left(\int_{-\pi}^{\pi} w'(\theta-\hat{\theta})\mathbf{r}^*(\hat{\theta})d\hat{\theta}\right)$$

where $\odot$ denotes the Hadamard product (i.e., element-wise multiplication), F' is the derivative of F with respect to its argument, and

$$w'(\theta-\hat{\theta}) = -\omega_0\sin(\theta-\hat{\theta}).$$

An input will cause a shift along the attractor if the projection of the input along this direction is nonzero. Since F is monotonically increasing, $F'(z) > 0$ for all z, and $1/a > 0$, so for the projection to be nonzero, we require only that the projection of the input onto

$$\int_{-\pi}^{\pi} w'(\theta-\hat{\theta})\mathbf{r}^*(\hat{\theta})d\hat{\theta} = \omega_0\int_{-\pi}^{\pi} \sin(\hat{\theta}-\theta)\mathbf{r}^*(\hat{\theta})d\hat{\theta}$$

is nonzero. Since the inputs **r***(θ - δ) and **r***(θ + δ) do not arrive at the same time, we can consider each input component individually to show the projection is non-zero. For the **r***(θ - δ) component, we have

$$\mathbf{r}^*(\theta-\delta)\cdot\omega_0\int_{-\pi}^{\pi}\sin(\hat{\theta}-\theta)\mathbf{r}^*(\hat{\theta})d\hat{\theta} = \omega_0\int_{-\pi}^{\pi}\int_{-\pi}^{\pi}\sin(\hat{\theta}-\theta)r^*(\hat{\theta})r^*(\theta-\delta)d\hat{\theta}d\theta$$

$$= \omega_0\int_{-\pi}^{\pi}\int_{-\pi}^{\pi}\left(\sin(\hat{\theta})\cos(\theta) - \cos(\hat{\theta})\sin(\theta)\right)r^*(\hat{\theta})r^*(\theta-\delta)d\hat{\theta}d\theta$$

$$= \omega_0\int_{-\pi}^{\pi}\cos(\theta)r^*(\theta-\delta)d\theta\int_{-\pi}^{\pi}\sin(\hat{\theta})r^*(\hat{\theta})d\hat{\theta} - \omega_0\int_{-\pi}^{\pi}\sin(\theta)r^*(\theta-\delta)d\theta\int_{-\pi}^{\pi}\cos(\hat{\theta})r^*(\hat{\theta})d\hat{\theta}$$

Since sin($\hat{\theta}$) is an odd function and **r***($\hat{\theta}$) is an even function, the integral of their product will be zero, so the first term above disappears and we are left with:

$$= -\omega_0\int_{-\pi}^{\pi}\sin(\theta)r^*(\theta-\delta)d\theta\int_{-\pi}^{\pi}\cos(\hat{\theta})r^*(\hat{\theta})d\hat{\theta}.$$

The second integral (over $\hat{\theta}$) is nonzero and positive, since **r***($\hat{\theta}$) is even and, from the shape of the bump, has a nonzero fundamental frequency in its Fourier expansion. The first integral (over *θ*) is also nonzero for δ nonzero because sin(*θ*) is an odd function and, from the shape of the bump and since **r***($\hat{\theta}$) is even, the shifted bump **r***(*θ* - *δ*) has a nonzero odd fundamental frequency component. Together, this gives a nonzero projection of the input onto the direction of the attractor, so that the input causes a shift in the bump.

In the discrete case, the input additionally must be active for sufficiently long so as to move the location of the bump to the location of the next evidence level.

The above argument also shows that the shift in the bump will be linear in the number of cues. This is because the stationary solutions are invariant under circular shifts. This means that the input will have the same form for any stationary solution, and hence the same shift along the attractor, provided that there is sufficient time between inputs for the solution to converge to the attractor.

Bump Maintains the Evidence Location Across Positions

When the position gate moves to position *i*, neuron $r_{i,j}$ of the active position layer receives input from the corresponding neuron $r_{i-1,j}$ of the previously active layer. Denote the evidence level of the peak of the bump by *B.* Since the bump at position *i*-1 has its greatest activity at *B*, neuron $r_{i,B}$ will have the greatest input. Since the bump of activity is a symmetrically shaped neutrally stable attractor, and the connectivity between layers *i*-1 and *i* is also symmetric, the bump at position *i* will also form a peak at *B*.

PLANAR BUMP ATTRACTOR

In Supplementary Figure 3, we present a model that accumulates velocity signals along the position axis to create the shifts in the active position while evidence continues to be integrated along the evidence axis, giving a planar bump attractor. The activity $r_{i,j}$ of a neuron at position *i* and evidence level *j* evolves according to

$$\frac{dr_{i,j}}{dt} = -ar_{i,j} + F\left(\sum_k W^{(e)}_{j,k} r_{i,k} + \sum_\ell \sum_m W^{(p)}_{i,\ell} r_{\ell,m} + c\left(I_{i,j+1,L}(t) + I_{i,j-1,R}(t)\right) + f\left(I_{i-1,j,v^+} + I_{i+1,j,v^-}\right)\right) \quad (S4)$$

,

where, as in the position-gated bump attractor, *a* is the exponential decay rate in the absence of input and *F* is the output nonlinearity. In this case, $W^{(e)}$ is the matrix of synaptic connections between neurons at the same position but different evidence levels and $W^{(p)}$ is the matrix of synaptic connections between neurons at different positions regardless of evidence level. $I_{i,j+1,L}$ and $I_{i,j-1,R}$ are the firing rates of the “shifter neurons” in evidence, which have synaptic connection strength *c*. $I_{i-1,j,v}$ and $I_{i+1,j,v}$ are the firing rates of the “shifter neurons” in position, which have synaptic connection strength *f*.

Parameterization of the Planar Bump Attractor

We took neurons at 17 different positions and 35 different evidence levels, for a total of 595 neurons. Each neuron was defined by an evidence angle, *θ*, and a position angle, *ϕ*. These angles were determined by assigning each evidence level an angle evenly spaced between 0 and 2*π*, and similarly assigning each position bin an angle evenly spaced between 0 and 2*π*. Along each axis, the strengths of synaptic connections were symmetric with excitatory

connections between neurons with similar tuning on that axis and inhibitory between neurons farther apart in their tuning. Specifically, the entries of the matrix of synaptic connections along the evidence axis $W^{(e)}$ for a neuron tuned to evidence *j* and a neuron tuned to evidence *k* are given by

$$W_{j,k}^{(e)} = \omega_0(\cos(\theta_j - \theta_k) + \omega_1)$$

and the entries of the matrix of synaptic connections along the position axis $W^{(p)}$ for a neuron tuned to position bin *i* and a neuron tuned to position bin *h* are given by

$$W_{i,h}^{(p)} = \omega_0(\cos(\phi_i - \phi_h) + \omega_1).$$

When an input is present, asymmetric connections result in a shift of the bump along the evidence axis in the direction of the input via a set of evidence shifter neurons,

$$I_{i,j,L}(t) = r_{i,j}\mathbf{1}_{\text{left}}(t)$$

and

$$I_{i,j,R}(t) = r_{i,j}\mathbf{1}_{\text{right}}(t)$$

where

$$\mathbf{1}_{\text{left}}(t) = \begin{cases} 1 & \min_{\ell \in L} |p(t) - \ell| < 0.5 \text{ cm} \\ 0 & \text{otherwise} \end{cases}$$

and

$$\mathbf{1}_{\text{right}}(t) = \begin{cases} 1 & \min_{R \in \mathcal{R}} |p(t) - R| < 0.5 \text{ cm} \\ 0 & \text{otherwise} \end{cases}$$

where *p*(*t*) is the position of the animal at time *t*, L is the set of positions of left cues and $\mathcal{R}$ is the set of positions of right cues.

Similarly, asymmetric input connections with a set of velocity shifter neurons result in shifts along the position axis, based on the animal's current velocity,

$$I_{i,j,v^+}(t) = r_{i,j}1_{v^+}(t)$$

and

$$I_{i,j,v^-}(t) = r_{i,j}1_{v^-}(t)$$

where

$$\mathbf{1}_{v^+}(t) = \begin{cases} \frac{|v(t)|}{50 \text{ cm/s}} & v(t) > 0 \\ 0 & \text{otherwise} \end{cases}$$

and

$$\mathbf{1}_{v^-}(t) = \begin{cases} \frac{|v(t)|}{50 \text{ cm/s}} & v(t) < 0 \\ 0 & \text{otherwise} \end{cases}$$

where *v*(*t*) is the animal's velocity at time *t*.

The neuronal nonlinearity is defined by

$$F(x) = \frac{q(1 + \tanh(\gamma x))}{2}.$$

The parameters used in the simulations are: $a = 55 \text{ s}^{-1}$, $q = 1250$ Hz/s, $\gamma = 1$ s/Hz, $\omega_0 = 0.12$, $\omega_1 = -1.0$, $c = 0.2 \text{ s}^{-1}$, $f = 0.045 \text{ s}^{-1}$. In Supplementary Fig. 3B,C, we used *v*(*t*) = 50 cm/s for

the entire maze. Neurons $r_{0,-1}$, $r_{0,0}$, and $r_{0,1}$ are initialized to 15 Hz, 17.5 Hz, and 15 Hz respectively, and all other neurons are initialized to 0 Hz activity. In Supplementary Fig. 3D, we used the same parameters but varied the velocity during different positions of the maze, taking

$$v(t) = \begin{cases} 50 \text{ cm/s} & t < 3.0 \text{ s} \\ -75 \text{ cm/s} & 3.0 \text{ s} \leq t < 4.5 \text{ s} \\ 0 \text{ cm/s} & 4.5 \text{ s} \leq t < 6.0 \text{ s} \\ 50 \text{ cm/s} & t \geq 6.0 \text{ s} \end{cases}.$$

Properties of the Planar Bump Attractor

The same arguments as for the position-gated bump attractor show that the bump will be stable along the evidence axis and shift linearly with evidence inputs. Similar arguments show that the bump will be stable along the position axis and that velocity will enact shifts in the bump location due to the connectivity along the position axis being analogous to that along the evidence axis. As in the case of the position-gated bump attractor, the location along the evidence axis will be maintained across positions since the neuron at the same evidence level will have the greatest input. Analogous arguments show that position will be maintained when evidence levels shift.

References

1. Pinto, L. *et al.* An accumulation-of-evidence task using visual pulses for mice navigating in virtual reality. *Front. Behav. Neurosci.* **12**, 36 (2018).
2. Skaggs, W. E., Knierim, J. J., Kudrimoti, H. S. & McNaughton, B. L. A model of the neural basis of the rat's sense of direction. *Adv. Neural Inf. Process. Syst.* **7**, 173–180 (1995).
3. Redish, A., Elga, A. & Touretzky, D. A coupled attractor model of the rodent head direction system. *Network* **7**, 671–685 (1996).
4. Zhang, K. Representation of spatial orientation by the intrinsic dynamics of the head-direction cell ensemble: a theory. *J. Neurosci.* **16**, 2112–2126 (1996).
5. Clark, B. J. & Taube, J. S. Vestibular and attractor network basis of the head direction cell signal in subcortical circuits. *Front. Neural Circuits* **6**, 7 (2012).
6. Ajabi, Z., Keinath, A. T., Wei, X.-X. & Brandon, M. P. Population dynamics of head-direction neurons during drift and reorientation. *Nature* **615**, 892–899 (2023).
7. Song, P. & Wang, X.-J. Angular Path Integration by Moving 'Hill of Activity': A Spiking Neuron Model without Recurrent Excitation of the Head-Direction System. *J. Neurosci.* **25**, 1002–1014 (2005).
8. Hulse, B. K. & Jayaraman, V. Mechanisms Underlying the Neural Computation of Head Direction. *Annu. Rev. Neurosci.* **43**, 31–54 (2020).
9. Turner-Evans, D. B. *et al.* The neuroanatomical ultrastructure and function of a biological ring attractor. *Neuron* **109**, 1582 (2021).
10. Kim, S. S., Hermundstad, A. M., Romani, S., Abbott, L. F. & Jayaraman, V. Generation of stable heading representations in diverse visual scenes. *Nature* **576**, 126–131 (2019).
11. Cope, A. J., Sabo, C., Vasilaki, E., Barron, A. B. & Marshall, J. A. R. A computational model of the integration of landmarks and motion in the insect central complex. *PLoS One* **12**, e0172325 (2017).
12. Green, J. *et al.* A neural circuit architecture for angular integration in Drosophila. *Nature*

546, 101–106 (2017).
13. Kim, S. S., Rouault, H., Druckmann, S. & Jayaraman, V. Ring attractor dynamics in the Drosophila central brain. *Science* **356**, 849–853 (2017).
14. McNaughton, B. L., Battaglia, F. P., Jensen, O., Moser, E. I. & Moser, M.-B. Path integration and the neural basis of the 'cognitive map'. *Nat. Rev. Neurosci.* **7**, 663–678 (2006).
15. Samsonovich, A. & McNaughton, B. L. Path integration and cognitive mapping in a continuous attractor neural network model. *J. Neurosci.* **17**, 5900–5920 (1997).
16. Conklin, J. & Eliasmith, C. A controlled attractor network model of path integration in the rat. *J. Comput. Neurosci.* **18**, 183–203 (2005).
17. Darshan, R., van Vreeswijk, C. & Hansel, D. Strength of Correlations in Strongly Recurrent Neuronal Networks. *Phys. Rev. X* **8**, 031072 (2018).
18. Noorman, M., Hulse, B. K., Jayaraman, V., Romani, S. & Hermundstad, A. M. Maintaining and updating accurate internal representations of continuous variables with a handful of neurons. *Nat. Neurosci.* **27**, 2207–2217 (2024).
19. Kishimoto, K. & Amari, S. Existence and stability of local excitations in homogeneous neural fields. *J. Math. Biol.* **7**, 303–318 (1979).
