# Supplementary material for: Neural circuit models for evidence accumulation through choice-selective sequences
Source: Nat Commun. 2026 Mar 17;17:4055. doi: 10.1038/s41467-026-70267-9 (PMC13139583; doi:10.1038/s41467-026-70267-9)
Supplement: Supplementary file 2 — Reporting Summary [file 41467_2026_70267_MOESM2_ESM.pdf]

nature portfolio

Corresponding author(s): Mark S. Goldman, Ilana B. Witten

Last updated by author(s): 1/9/2026

Reporting Summary

Nature Portfolio wishes to improve the reproducibility of the work that we publish. This form provides structure for consistency and transparency in reporting. For further information on Nature Portfolio policies, see our Editorial Policies and the Editorial Policy Checklist.

Statistics

For all statistical analyses, confirm that the following items are present in the figure legend, table legend, main text, or Methods section.

n/a	Confirmed	
☐	☒	The exact sample size (*n*) for each experimental group/condition, given as a discrete number and unit of measurement
☐	☒	A statement on whether measurements were taken from distinct samples or whether the same sample was measured repeatedly
☐	☒	The statistical test(s) used AND whether they are one- or two-sided<br>*Only common tests should be described solely by name; describe more complex techniques in the Methods section.*
☐	☒	A description of all covariates tested
☐	☒	A description of any assumptions or corrections, such as tests of normality and adjustment for multiple comparisons
☐	☒	A full description of the statistical parameters including central tendency (e.g. means) or other basic estimates (e.g. regression coefficient) AND variation (e.g. standard deviation) or associated estimates of uncertainty (e.g. confidence intervals)
☐	☒	For null hypothesis testing, the test statistic (e.g. *F*, *t*, *r*) with confidence intervals, effect sizes, degrees of freedom and *P* value noted<br>*Give P values as exact values whenever suitable.*
☒	☐	For Bayesian analysis, information on the choice of priors and Markov chain Monte Carlo settings
☒	☐	For hierarchical and complex designs, identification of the appropriate level for tests and full reporting of outcomes
☐	☒	Estimates of effect sizes (e.g. Cohen's *d*, Pearson's *r*), indicating how they were calculated

Our web collection on statistics for biologists contains articles on many of the points above.

Software and code

Policy information about availability of computer code

Data collection	Virtual reality behavioral data was collected using Matlab code (Matlab 2015b and above, Mathworks Inc) running the ViRMEn package (https://pni.princeton.edu/pni-software-tools/virmen-virtual-reality-matlab-engine), and using the following Arduino based optical sensor package (https://github.com/sakoay/AccumTowersTools/tree/master/OpticalSensorPackage). Experimental code is collected at the following public repository (https://github.com/BrainCOGS/TankMouseVR). ACC and DMS imaging data were processed with Suite2p. Spike inference was performed based on the algorithm from Jewell et al. (2020) (https://github.com/jewellsean/FastLZeroSpikeInference).

Data analysis

Data analysis was performed using custom python code (anaconda python 3.6.10) and is publicly available on GitHub (https://github.com/lindseysbrown/evidence_accumulation_through_sequences). Additional source code for VR behavioral analysis can be found at https://github.com/BrainCOGS/behavioralAnalysis.

Custom code uses the following python packages:
h5py==2.10.0
matplotlib==3.3.4
numpy==1.19.2
pandas==1.1.5
scikit_learn==1.2.2
scipy==1.5.2
seaborn==0.11.1
cebra (https://cebra.ai/docs/installation.html)

For manuscripts utilizing custom algorithms or software that are central to the research but not yet described in published literature, software must be made available to editors and reviewers. We strongly encourage code deposition in a community repository (e.g. GitHub). See the Nature Portfolio guidelines for submitting code & software for further information.

Data

Policy information about availability of data

All manuscripts must include a data availability statement. This statement should provide the following information, where applicable:

- Accession codes, unique identifiers, or web links for publicly available datasets
- A description of any restrictions on data availability
- For clinical datasets or third party data, please ensure that the statement adheres to our policy

Code for the simulation of all models and the data analysis is available at https://github.com/lindseysbrown/evidence_accumulation_through_sequences. Data from HPC is previously published and available (Nieh et al. (2021), https://www.nature.com/articles/s41586-021-03652-7#Sec33). Data from RSC is previously published and available (Koay, S. A. Sequential and efficient neural-population coding of complex task information. (2021) doi:10.5061/dryad.cvdncjt53.). Full datasets used for analysis in this work from ACC, DMS, and HPC are available (https://doi.org/10.6084/m9.figshare.30921038).

Research involving human participants, their data, or biological material

Policy information about studies with human participants or human data. See also policy information about sex, gender (identity/presentation), and sexual orientation and race, ethnicity and racism.

Reporting on sex and gender	N/A
Reporting on race, ethnicity, or other socially relevant groupings	N/A
Population characteristics	N/A
Recruitment	N/A
Ethics oversight	N/A

Note that full information on the approval of the study protocol must also be provided in the manuscript.

Field-specific reporting

Please select the one below that is the best fit for your research. If you are not sure, read the appropriate sections before making your selection.

☒ Life sciences ☐ Behavioural & social sciences ☐ Ecological, evolutionary & environmental sciences

For a reference copy of the document with all sections, see nature.com/documents/nr-reporting-summary-flat.pdf

Life sciences study design

All studies must disclose on these points even when the disclosure is negative.

Sample size	Statistical calculations were not used to select sample sizes. Number of animals per group were chosen based on sample sizes in comparable tasks (for example, Yartsev et al 2018; Deverett et al 2019) and on the availability of animals.
Data exclusions	For some analysis, trial exclusions were implemented and are fully described in Methods, Trial Selection Criteria. These trials were trials which were "warm up" trials (not in the accumulation of evidence task level) and trials with excessive travel.
Replication	We report results from multiple mice for each region, which consisted of multiple cohorts of mice. Results were consistent across mice.

Randomization	Animals were not randomly allocated to recording groups. Data for each recording site came from separate experiments. There were not multiple experimental conditions per recording site to randomize.
Blinding	Experimenters were not blinded to recording groups. Recordings from different regions were performed by different experimenters, making them aware of the region they are recording from. Automated analyses were used wherever possible. We applied the same analyses to each brain region to produce tuning curves and population summary plots (Fig. 5, Supplementary Figs. 5,12) in order to draw conclusions about the monotonicity of each region.

Reporting for specific materials, systems and methods

We require information from authors about some types of materials, experimental systems and methods used in many studies. Here, indicate whether each material, system or method listed is relevant to your study. If you are not sure if a list item applies to your research, read the appropriate section before selecting a response.

Materials & experimental systems

n/a	Involved in the study
☒	☐ Antibodies
☒	☐ Eukaryotic cell lines
☒	☐ Palaeontology and archaeology
☐	☒ Animals and other organisms
☒	☐ Clinical data
☒	☐ Dual use research of concern
☒	☐ Plants

Methods

n/a	Involved in the study
☒	☐ ChIP-seq
☒	☐ Flow cytometry
☒	☐ MRI-based neuroimaging

Animals and other research organisms

Policy information about studies involving animals; ARRIVE guidelines recommended for reporting animal research, and Sex and Gender in Research

Laboratory animals	Three female mice of CaMKIIa-tTA (JAX 007004) crossed with tetO-GCaMP6s130 (JAX 024742) were used for two-photon imaging of ACC. D1R-Cre (n = 3, female, EY262Gsat, MMRRC-UCD), A2a-Cre (n = 3, male, KG139Gsat, MMRRC-UCD), and D2R-Cre (n = 2, male, ER44Gsat, MMRRC-UCD) mice were used for DMS two-photon imaging. Mice underwent surgery at 3-4 months and training and imaging continued for an additional 3-4 months.
Wild animals	The study did not involve wild animals.
Reporting on sex	Data was included from both sexes. Analyses were not separated by sex.
Field-collected samples	This study did not involve samples collected from the field.
Ethics oversight	All procedures were conducted in accordance with National Institute of Health guidelines and were reviewed and approved by the Institutional Animal Care and Use Committee at Princeton University.

Note that full information on the approval of the study protocol must also be provided in the manuscript.

Plants

Seed stocks	*Report on the source of all seed stocks or other plant material used. If applicable, state the seed stock centre and catalogue number. If plant specimens were collected from the field, describe the collection location, date and sampling procedures.*
Novel plant genotypes	*Describe the methods by which all novel plant genotypes were produced. This includes those generated by transgenic approaches, gene editing, chemical/radiation-based mutagenesis and hybridization. For transgenic lines, describe the transformation method, the number of independent lines analyzed and the generation upon which experiments were performed. For gene-edited lines, describe the editor used, the endogenous sequence targeted for editing, the targeting guide RNA sequence (if applicable) and how the editor was applied.*
Authentication	*Describe any authentication procedures for each seed stock used or novel genotype generated. Describe any experiments used to assess the effect of a mutation and, where applicable, how potential secondary effects (e.g. second site T-DNA insertions, mosiacism, off-target gene editing) were examined.*
